# MiR-374b-5p Regulates T Cell Differentiation and Is Associated with rEg.P29 Immunity

**DOI:** 10.1155/2020/8024763

**Published:** 2020-08-21

**Authors:** Dongjie Li, Xiancai Du, Mingxing Zhu, Songhao Yang, Wei Zhao

**Affiliations:** ^1^Echinococcosis Laboratory, Ningxia Medical University, Yinchuan, Ningxia, China; ^2^Department of Clinical Laboratory, People's Hospital of Ningxia Hui Autonomous Region, Yinchuan, Ningxia, China

## Abstract

*Cystic echinococcosis* (CE) is a zoonotic disease caused by *Echinococcus granulosus* (Eg) infection. Our previous study confirmed that recombinant Eg.P29 (rEg.P29) could protect against *echinococcus granulosus* secondary infection in sheep and mice. The aim of the study was to investigate the association between immunoprotection of rEg.P29 vaccine and mmu-miR-374b-5p (miR-374b-5p) and study the immunity influence of miR-374b-5p on CD4^+^ T cells in mice spleen. MiR-374b-5p level was significantly increased after the second-week and the fourth week of vaccination with rEg.P29. Overexpression of miR-374b-5p increased IFN-*γ*, IL-2, IL-17A mRNA levels and decreased IL-10 mRNA levels in CD4^+^ T cells. Moreover, the inhibition of miR-374b-5p decreased IFN-*γ* and IL-17A and increased IL-10 mRNA levels in CD4^+^ T cells; this was further confirmed by the flow cytometry. The vaccination of rEg.P29 enhanced miR-374b-5p expression that was associated with a higher Th1 and Th17 immune response, a lower IL-10 mRNA production with miR-374b-5p overexpression, a lower Th1 immune response, and a higher IL-10 mRNA levels with miR-374b-5p inhibitions. To sum up, these data suggest that miR-374b-5p may participate in rEg.P29 immunity by regulating Th1 and Th17 differentiation.

## 1. Introduction

*Cystic echinococcosis* (CE) is a very severe zoonotic helminthic disease, which may be fatal if the disease are not appropriately diagnosed and subsequently treated [1]. It is a kind of neglected disease estimated to be responsible for the yearly loss of one million disability-adjusted life years [2]. This zoonotic disease is caused by *cestodes* of the genus *Echinococcus* [3, 4]. An increasing understanding of immunological events that account for the metacestode survival in human and murine CE infection prompted numerous explorative experiments tackling the potential of novel preventive measures [2, 3, 5]. The infection with *echinococcus granulosus* triggers an immune response that is characterized by an imbalance between an initial cellular (Th1) and a subsequently prevailing humoral (Th2) immune response [1]. The current evidence suggests that protection against *E. multilocularis* infection is basically associated with the maintenance of a Th1-oriented cellular immune response, while an increasingly dominating Th2 profile has been associated with a rather progressive form of *alveolar echincoccosis* (AE) in humans [6]. Th17 cells have an essential role in inflammatory pathology and antiparasitic immunity. Previous data have shown that IL-17A is produced during human *cystic echinococcosis* and is involved in the host defense mechanisms against the extracellular parasite *E. granulosus* [7]. Existing studies have suggested a beneficial, preventive effect of IL-17A and IFN-*γ* against *E. granulosus* in humans. Th17 and Th1 cells may have complementary roles in protection against CE, with both being indispensable for complete protection [8, 9].

Immunoprophylaxis is an ideal way to prevent the epidemic of CE, especially in the early stage of the disease. In *echinococcus granulosus* infection, some vaccine candidates have proven to be highly protective in mice [5, 10, 11]. Nevertheless, due to complicated multicellular pathogen and host interplay, there is still no approved vaccine for clinical use. The P29 protein was first identified by Gonzalez et al. as a novel 29 kDa antigen from *E. granulosus protoscoleces*, while looking for parasite antigens distinct from those present in *hydatid cyst* fluid. Subsequently, the same protein (EgP29) has been characterized within *echinococcosis granulosus* sensu stricto, protoscolex-derived soluble somatic antigen, as a biomarker for monitoring CE patients [12]. Prior studies suggested a possible role of rEg.P29 as a developmentally regulated component of the *E*. *granulosus metacestode* [6]. Our previous study confirmed that recombinant Eg.P29 (rEgP29) could protect against *echinococcus granulosus* secondary infection in sheep and mice [13, 14]. Immunization with rEg.P29 induced protective immunity against sheep carrying natural infection of *E. granulosus* eggs [5]. Vaccination of mice with bacterially produced rEgP29 resulted in significant protective immunity, inducing 96.6% protection against challenge infection with *E. granulosus protoscoleces* [4]. Previous studies have demonstrated that CD4^+^ Th cell subpopulations are altered when infected with *E. granulosus*, and importantly, the Th1 immune response characterized by interferon-gamma (IFN-*γ*) is associated with protection against *E. granulosus* infection [5]. Although the innate immune responses favoring Th1 type immunity are produced following rEgP29 immunization, the molecular determinants of such responses remain unknown.

MicroRNAs (miRNAs), a class of small regulatory RNAs, are involved in the regulation of many biological processes, primarily through the repression of messenger RNAs (mRNAs) that typically binds to the 3′ untranslated region (3′UTR) of target genes [15]. miRNAs have been shown to regulate multiple functions of T cell subsets, including their development, survival, and activation in humans and mice [16–19]. The existing studies on miRNAs in *cestodes* have mainly focused on miRNA predictions of target genes, which provided a platform for the identification of miRNAs using both computational and experimental approaches. However, there is still a lack of comprehensive functional and experimental validation of these putative miRNA targets [20]. Studying the role of miRNAs in parasite immunity still remains a challenge [21]. Mmu-miR-374b-5p (MiR-374b-5p), a highly conserved miRNA, has shown to be involved in different physiological and pathological processes [22–26]. Over recent years, studies have indicated that miR-374b-5p can accelerate cell proliferation and the production of aberrant glycosylated immunoglobulin (Ig)A1 in B cells and inhibit proliferation and promote apoptosis of T cell lymphoblastic lymphoma [22, 24], while its function in vaccine immunity to *echinococcus* infection remains unclear. We used a miRNA microarray to assess miRNA expression in CD4^+^ T cells of rEg.P29 vaccination of *echinococcus granulosus* secondary murine infection model, where bioinformatics analysis revealed that miR-374b-5p was significantly differentially increased. Furthermore, we examined the influence of miR-374b-5p on the function of naive CD4^+^ T cell differentiation.

## 2. Materials and Methods

### 2.1. Animals

Female C57/BL6 mice, 6–8 weeks old, were obtained from the Animal Experimentation Center of Ningxia Medical University (Yinchuan, China). All the animals were housed in an environment with a temperature of 22 ± 1°C, relative humidity of 50 ± 1%, and a light/dark cycle of 12/12 hr. All animal studies (including the mice euthanasia procedure) were done in compliance with the regulations and guidelines of Ningxia Medical University institutional animal care and conducted according to the AAALAC and the IACUC guidelines.

### 2.2. Vaccination of Mice

Eg.P29 gene was taken from *hydatid cysts* of patients in the General Hospital of Ningxia Medical University (the Chinese strain of the gene was recorded into GeneBank: sequence number AF078931) [9]. Plasmid Eg.P29/pET28a was previously constructed and expressed in Escherichia coli at our laboratory [13, 14]. The purified rEg.P29 was identified with 12% sodium dodecyl sulfate-polyacrylamide gel electrophoresis (SDS-PAGE) and Western blot [8].

Twenty female C57/BL6 mice (6–8 weeks old) were randomly allocated into two groups of 10 mice each. Mice in rEg.P29 immunity group were subcutaneously immunized with 10 mg of rEgP-29 in 100-ml PBS emulsified in Freund's adjuvant that was injected in the intraperitoneal cavity. The mice in group control were injected with adjuvant PBS. One, 2, 3, 4, 5, and 6 weeks after injection, mice were euthanized by cervical dislocation.

### 2.3. T Cell Isolation, CD4^+^ T Cell Sorting

Spleens were aseptically removed and placed in a sterile Roswell Park Memorial Institute (RPMI) 1640 medium (Sigma) supplemented with 10% fetal bovine serum (FBS), penicillin (1 × 10^5^ units/L), and streptomycin (100 mg/L) (Sigma) from rEgP-29 vaccinated mice and controls. The lymphocytes were obtained using the spleen lymphocyte cell separation medium kit (Solarbio Life Sciences, P.R. China) according to the manufacturer's instructions. Subsequently, CD4^+^ T cells were isolated using magnetic-activated cell separation (MACS) and a mouse CD4^+^ T Cell Isolation Kit (Miltenyi Biotec, Germany) (purity was >90% as checked by flow cytometry) according to the manufacturers' instructions. CD4^+^ T cells were cultured in RPMI 1640 medium at 37°C in 5% CO_2_ with 10% fetal bovine serum and 100 U/ml concentrations of penicillin and streptomycin.

### 2.4. QRT-PCR

Total RNA was extracted from CD4^+^T cells of spleen using Trizol reagent (Invitrogen) according to the manufacturer's procedures. For miRNA-specific reverse transcription, the reaction was performed using the Mir-X™ miRNA First-Strand Synthesis Kit (Takara) and reverse transcription primers from Mir-X™ miRNA First-Strand Synthesis Kit (Takara) on the ABI 7500 fast real-time PCR system (Applied Biosystems, Foster City, CA, USA), according to the manufacturer's instructions. The miR-374b-5p PCR primers were purchased from Qiagen (218300). U6 was utilized as an internal control. U6, forward: 5′-CTCGCTTCGGCAGCACA-3′; reverse: 5′-AACGCTTCACGAATTTGCGT-3′. The relative miRNA levels were calculated using the 2 ^–*ΔΔ*Ct^ method.

### 2.5. Transfections

Mouse naive CD4^+^ T cells (naive CD4^+^ CD62L^+^ T cell Isolation Kit II, Miltenyi Biotech) were cultured at 37°C (5%CO_2_) in complete 1640 medium in 1 × 10^6^ cells/well of 96-well plates and were then transfected with mmu-miR-374b-5p mimics sequences at 50 nmol/ml (Qiagen,219600) and inhibitors sequences at 200 nmol/ml (Qiagen,219300) using Hiperfect Transfection Reagent (Qiagen) according to the manufacturer's protocols. After 4 h, the transfected cells were treated with anti-CD3 (1 *μ*g/ml) and anti-CD28 (1 *μ*g/ml) (eBioscience) for 48 h at 37°C in 48-well plates. For transfection, miScript negative control siRNA sequence (Qiagen,1027271) was used as a control. Total RNA was extracted from transfected cells using Trizol reagent (Invitrogen) according to the manufacturer's instructions and the levels of gene expression were measured by real-time RT-PCR. Reverse transcription-quantitative polymerase chain reaction was performed using the PrimeScript™ RT Master Mix (Takara) and TB Green™ Advantage qPCR Premix (Takara). The murine PCR primers were designed by the use of the Primer-BLAST tool on the NCBI website. IFN-*γ*: (forward) GCCACGGCACAGTCATTGA; (reverse) TGCTGATGGCCTGATTGTCTT; IL-2: (forward) TGAGCAGGATGGAGAATTACAGG; (reverse) GTCCAAGTTCATCTTCTAGGCAC; IL-4: (forward) ATCATCGGCATTTTGAACGAGG; (reverse) TGCAGCTCCATGAGAACACTA; IL-17A: (forward) TTTAACTCCCTTGGCGCAAAA; (reverse) CTTTCCCTCCGCATTGACAC; IL-12a: (forward) AGACATCACACGGGACCAAAC; (reverse) CCAGGCAACTCTCGTTCTTGT; Foxp3: (forward) ACCATTGGTTTACTCGCATGT; (reverse) TCCACTCGCACAAAGCACTT; IL-10: (forward) AGCCTTATCGGAAATGATCCAGT; (reverse) GGCCTTGTAGACACCTTGGT. All mRNA expressions were normalized to GAPDH gene expression. GAPDH, forward: 5′-CCATGTTTGTGATGGGTGTG-3′; reverse: 5′-CCTTCTTGATGTCATCATAC-3; the qPCR results were calculated using the 2^–*ΔΔ*Ct^ method.

### 2.6. T Cell Differentiation

Naive CD4^+^T cells (5 × 10^5^ cells/well) were cultured at 37°C (5% CO_2_) in complete 1640 medium in each well of 96-well plates and were then transfected with mmu-miR-374b-5p mimics 50 nmol/ml (Qiagen) and mmu-miR-374b-5p inhibitors 200 nmol/ml(Qiagen) using Hiperfect Transfection Reagent (Qiagen) according to the manufacturer's protocols. MiScript negative control siRNA sequence (Qiagen) was used as a control. After 4 h, the transfected cells were differentiated to four subtypes of T cells, Th1, Th2, Th17, and T regulatory cells using four different cytokine regimens in 48-well plates. Then, we followed the methods of Farshid Noorbakhsh et al. 2017 [27]. For Th1 cells, transfected cells were cultured in complete RPMI, plate-bound CD3 antibody (1 *μ*g/ml), and soluble CD28 antibody (1 *μ*g/ml), IL-2 (20 ng/ml), IL-12 (50 ng/ml), and anti-IL-4 antibody (10 ng/ml) (BD Biosciences) for 96 h. For Th2, the cells were treated with 1 *μ*g/ml plate-bound CD3 antibody, 1 *μ*g/ml anti-CD28, 20 ng/ml IL-2, 10 ng/ml IL-4 (BD Biosciences), and 10 ng/ml anti-IFN-*γ* for 96 h. To differentiate the cells into Th17 cells, transfected cells were cultured in complete RPMI, plate-bound CD3 antibody (1 *μ*g/ml), and soluble CD28 antibody (0.2 *μ*g/ml), TGF-*β* (5 ng/ml), IL-6 (100 ng/ml), anti-IFN-*γ* (10 ng/ml), anti-IL-4 (10 ng/ml), and IL-23 (50 ng/ml) (BD Biosciences) for 96 h. For regulatory T (Treg) cells, transfected cells were cultured in complete RPMI, plate-bound CD3 antibody (1 *μ*g/ml), and soluble CD28 antibody (0.2 *μ*g/ml), IL-2 (20 ng/ml), and TGF-*β*1 (50 ng/ml) (BD Biosciences) for 96 h.

### 2.7. Flow Cytometry

For intracellular staining, cells were stimulated with Cell Stimulation Cocktail plus Protein Transport Inhibitors (Invitrogen) for 6 h before staining. To detect the intracellular expression of interferon (IFN)-*γ*, interleukin (IL)-4, interleukin (IL)-17A, and FOXP3 in transfected CD4^+^ T cells, cells (0.5 × 10^6^/wells) were subjected to intracellular cytokine staining using a Cell Fixation/Permeabilization Kit (Invitrogen) following the manufacturer's instructions. Cells were stained with anti-CD4 and anti-CD3 antibodies and then fixed with Invitrogen's Fixation Buffer for 30 min at 4°C in darkness. Cells were permeabilized with Invitrogen's Permeabilization Buffer (1x) and then stained with flurochrome-conjugated anti-IFN-*γ*, anti-IL-4, anti-IL-17A, and anti-Foxp3, anti-CD25 antibodies (BD Biosciences) [27]. Stained cells were assayed with a BD FACSCalibur flow cytometer (BD Biosciences), and results were analyzed with FlowJoXsoftware (Tree star, Inc.).

### 2.8. Statistical Analysis

All statistical analyses were performed using Prism 7.0 (GraphPad Software). Data were obtained from at least three independent experiments and represented as mean ± standard error. Statistical analysis was conducted using the Student *t*-test or the Mann–Whitney *U* tests. Results were considered significant at *P* < 0.05.

## 3. Results

### 3.1. Increased Expression of miR-374b-5p Was Validated in rEgP-29-Vaccinated CD4^+^ T Cell of Spleens

**Figure 1:**
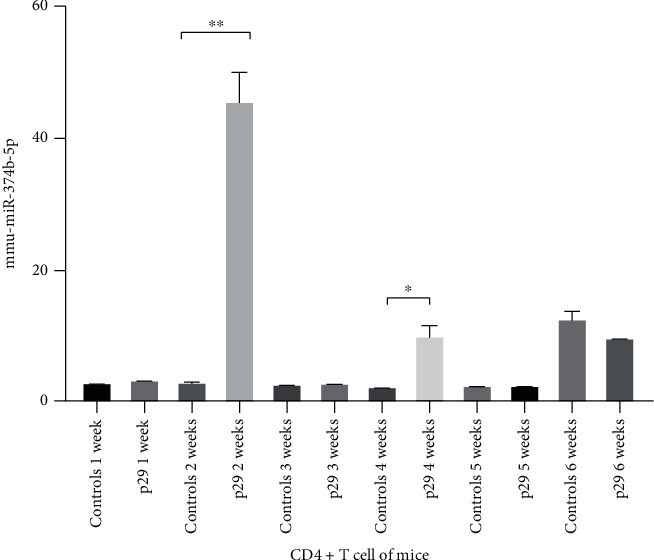
Expression of mmu-miR-374b-5p in CD4^+^ T cell from rEgP-29-vaccinated mice and controls. ^∗^*P* < 0.05; ^∗∗^*P* < 0.01.

To measure the expression of miR-374b-5p in CD4^+^ T cell of rEgP-29-vaccinated mice and controls, a quantitative real-time assay was used to examine the expression of miR-374b-5p. The obtained results revealed that miR-374b-5p was statistically increased by 16.7 fold-change after the second week in rEgP-29-vaccinated mice compared with control mice and was increased by 4.9 fold-change after the fourth week compared with controls (Figure 1). MiR-374b-5p may have a transient role in rEg.P29 immunity response.

### 3.2. MiR-374b-5p Mimics Upregulated IFN-*γ*, IL-2, IL-17A mRNA Expression and Downregulated IL-10 mRNA Expression in CD4^+^ T Cells. MiR-374b-5p Inhibitors Downregulated IFN-*γ*, IL-17A mRNA Expression, Upregulated IL-10 and IL-12a mRNA Expression in CD4^+^ T Cell

**Figure 2:**
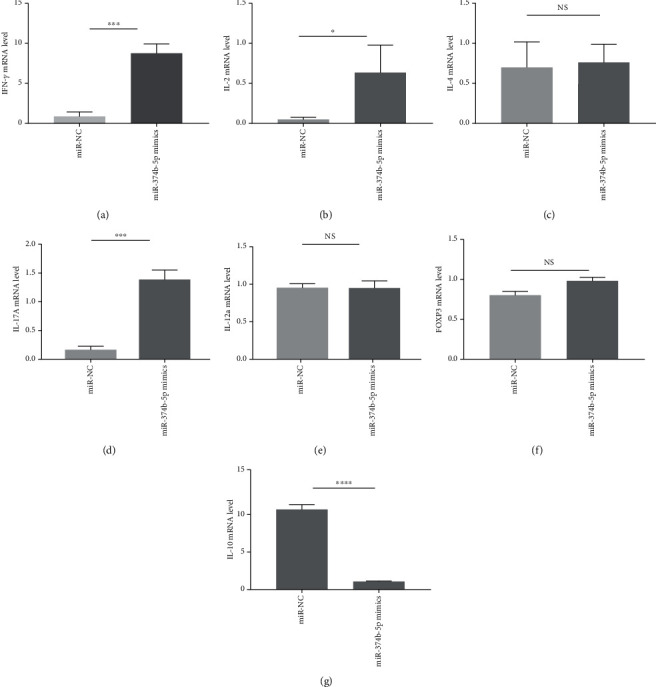
Naive CD4^+^ T cells were activated with plate-bound anti-CD3 (1 *μ*g/ml) and anti-CD28 (1 *μ*g/ml) in the presence of transfected mmu-miR-374b-5p mimics in vitro. 48 hours later, IFN-*γ*, IL-2, IL-4, IL-17A, IL-12a, FOXP3, and IL-10 mRNA expressions were analyzed by quantitative qRT-PCR. (a) IFN-*γ* mRNA, (b) IL-2 mRNA, (c) IL-4 mRNA, (d) IL-17A mRNA, (e) IL-12a mRNA, (f) FOXP3, and (g) IL-10 mRNA levels. Data from three independent experiments are shown (NS: not significant; ^∗^*P* < 0.05; ^∗∗^*P* < 0.01; ^∗∗∗^*P* < 0.001).

**Figure 3:**
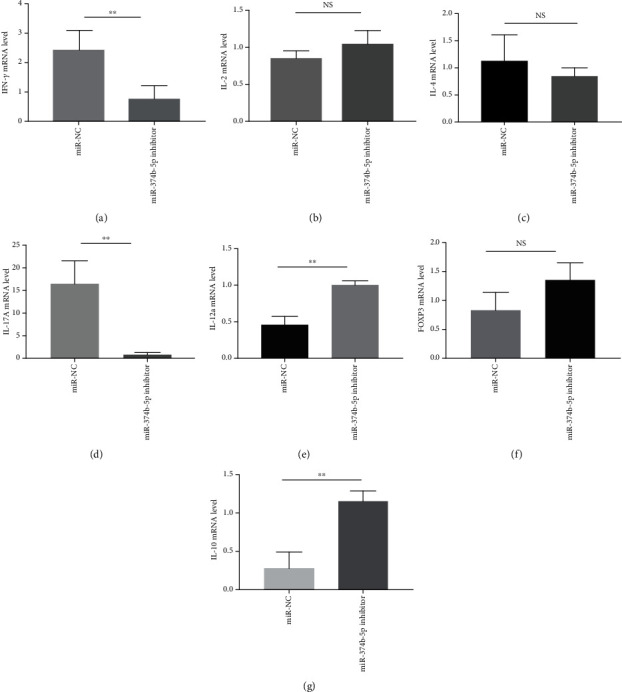
Naive CD4^+^ T cells were activated with plate-bound anti-CD3 (1 *μ*g/ml) and anti-CD28 (1 *μ*g/ml) in the presence of transfected mmu-miR-374b-5p inhibitors in vitro. 48 hours later, IFN-*γ*, IL-2, IL-12a, IL-4, IL-10, IL-17A, and FOXP3 mRNA expressions was analyzed by quantitative qRT-PCR. (a) IFN-*γ* mRNA, (b) IL-2 mRNA, (c) IL-4 mRNA, (d) IL-17A mRNA, (e) IL-12a mRNA, (f) FOXP3, and (g) IL-10 mRNA levels. Data from three independent experiments are shown (NS: not significant; ^∗^*P* < 0.05; ^∗∗^*P* < 0.01; ^∗∗∗^*P* < 0.001).

To detect the expression of IFN-*γ*, IL-2, IL-12a, IL-4, IL-10, IL-17A, and FOXP3 in CD4^+^ T cell of miR-374b-5p mimics treated cells, the qRT-PCR assay was performed (Figure 2). The mRNA levels of IFN-*γ*, IL-2, and IL-17A were statistically upregulated by 10.1, 13.7, and 8.5 fold-change in miR-374b-5p mimics treated cells compared with negative controls, respectively (Figures 2(a), 2(b), and 2(d)), and IL-10 mRNA levels were statistically downregulated (Figure 2(g)) by 9.9 fold-change in miR-374b-5p mimics treated cells compared with negative controls(all *P* < 0.05); however, there was no statistical difference in IL-4, IL-12a, and FOXP3 (Figures 2(c), 2(e), and 2(f)). Moreover, the mRNA levels of IFN-*γ* and IL-17A were significantly downregulated by 3.2 and 22.0 fold-change in miR-374b-5p inhibitors treated cells compared with negative controls, respectively (Figures 3(a) and 3(d)), and the mRNA levels of IL-12a and IL-10 were significantly upregulated by 2.2 and 4.1 fold-change in miR-374b-5p inhibitors treated cells compared with negative controls, respectively (Figures 3(e) and 3(g); all *P* < 0.05). IL-2, IL-4, and FOXP3 were not significantly different (Figures 3(b), 3(c), and 3(f)).

### 3.3. MiR-374b-5p Mimics Induced Differentiation of Naive CD4^+^ T Cells towards Th1 Subset and Th17 Subset, miR-374b-5p Inhibitors Suppressed Differentiation of Naive CD4^+^ T Cells towards Th1 Subset

**Figure 4:**
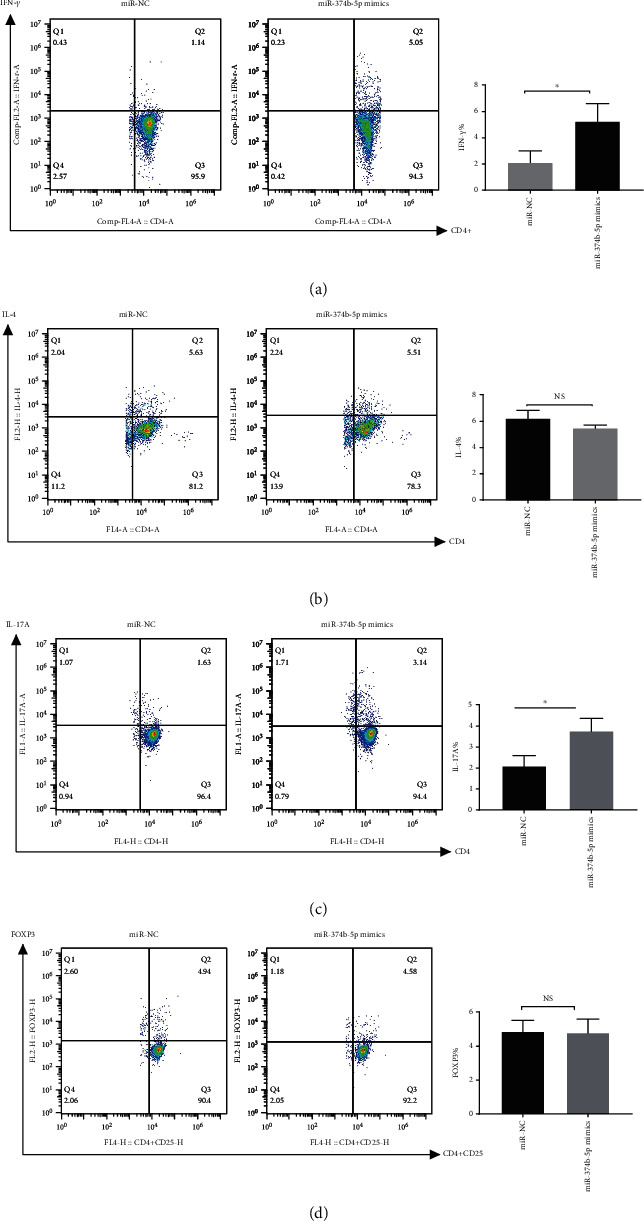
Overexpression of miR-374b-5p affects the differentiation of CD4^+^ T cells. Naive CD4^+^ T cells were isolated from mouse splenocytes (a) miR-374b-5p mimics, and negative control sequences were transfected into CD4^+^ T cells, which were activated and polarized under Th1 cytokine regimens. Representative dot plots and the percentages of IFN-*γ* within the CD4^+^ T cells. (b) Representative dot plots and the percentages of IL-4 within the CD4^+^ T cells, which were activated and polarized under Th2 cytokine regimens. (c) Representative dot plots and the percentages of IL-17A within the CD4^+^ T cells, which were activated and polarized under Th17 cytokine regimens. (d) Representative dot plots and the percentages of FOXP3 within the CD4^+^ T cells, which were activated and polarized under Treg cytokine regimens. Data are shown from 3 independent experiments. Data are from a single experiment representative of three independent experiments (NS: not significant; ^∗^*P* < 0.05).

**Figure 5:**
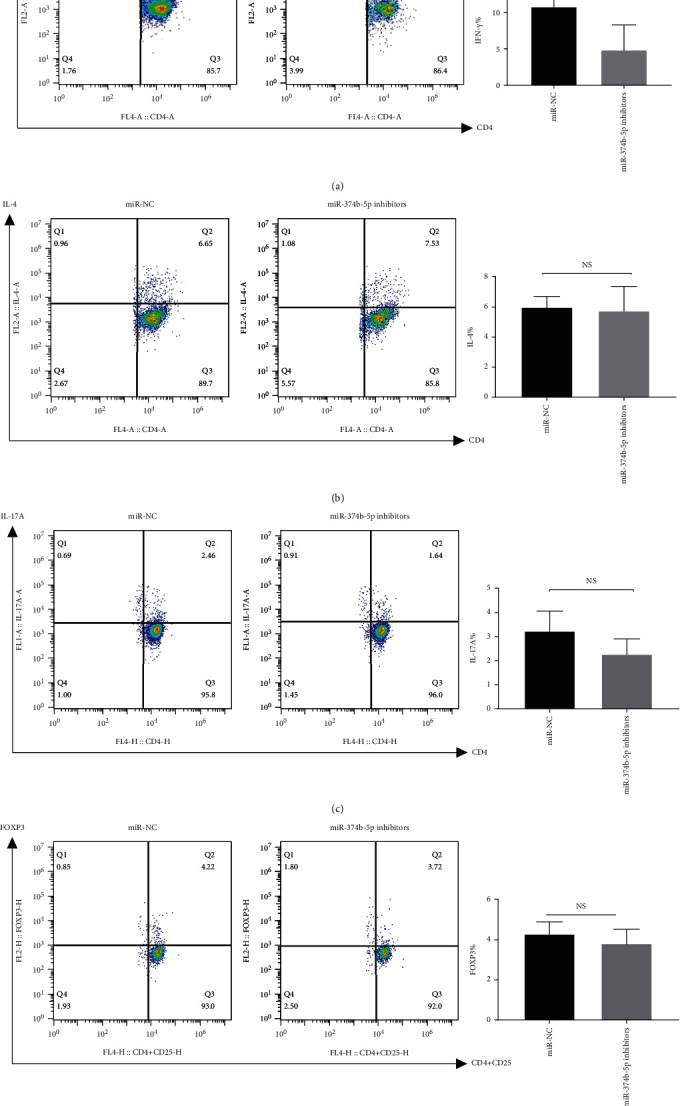
Inhibition of miR-374b-5p naive CD4 ^+^ T cells was isolated from mouse splenocytes. (a) miR-374b-5p inhibitors and negative control sequences were transfected into CD4^+^ T cells, which were activated and polarized under Th1 cytokine regimens. Representative dot plots and the percentages of IFN-*γ* within the CD4^+^ T cells. (b) Representative dot plots and the percentages of IL-4 within the CD4^+^ T cells, which were activated and polarized under Th2 cytokine regimens. (c) Representative dot plots and the percentages of IL-17A within the CD4^+^ T cells, which were activated and polarized under Th17 cytokine regimens. (d) Representative dot plots and the percentages of FOXP3 within the CD4^+^ T cells, which were activated and polarized under Treg cytokine regimens. Data are shown from 3 independent experiments. Data are from a single experiment representative of three independent experiments. (NS, not significant; ^∗^*P* < 0.05).

Considering the upregulation of IFN-*γ*, IL-2, and IL-17A mRNA levels in anti-CD3/CD28 activated naive CD4^+^ T cells, we investigated the effect of miR-374b-5p on CD4^+^ T cell differentiation. Purified CD4^+^ T cells were transfected with miR-374b-5p mimics, after which the inhibitors were cultured in the presence of anti-CD3 and anti-CD28 in the presence of the required cytokines for differentiation to Th1, Th2, Th17, and Treg cells. The results showed increased differentiation of miR-374b-5p mimics transfected cells towards IFN-*γ* producing Th1 subtype, compared with T cells transfected with a control miRNA sequence (Figure 4(a)). There was also a significant increase in the frequency of IL-17A-producing Th17 cells compared with T cells transfected with a control miRNA sequence (Figure 4(c)). A significant decrease in the frequency of IFN-*γ*-producing Th1 cells in miR-374b-5p inhibitors transfected T cells was also observed compared with a control miRNA sequence (Figure 5(a)). The frequency of Th2 and Treg cells did not show any significant difference following miR-374b-5p transfection (Figures 4(b), 4(d), 5(b), and 5(d)). There was also a mild decrease of IL-17A-producing Th17 cells in miR-374b-5p inhibitors transfected cells compared with T cells transfected with a control miRNA sequence; however, the decrease of IL-17A did not reach statistical significance (Figure 5(c)). Taken together, these data may suggest that miR-374b-5p promotes naive CD4^+^ T cell differentiation into Th1 cells.

## 4. Discussion

CE, which is a serious and potentially fatal disease for humans and animals, is still without effective prevention strategy [28]. Therefore, the development of a vaccine for early immunoprotection is required for the prevention of CE [29]. Previous studies have shown that rEg.P29 can induce protective immunity in mice against secondary infection of *echinococcus granulosus*. It has also been shown that cellular immunity is further polarized into Th1 after vaccination of rEg.P29 [6, 13]. So far, the core biological function of rEg.P29 in echinococcus biology is still unclear. Therefore, the future functional characterization of rEg.P29 may further the understanding of the molecular mechanism associated with rEg.P29. MicroRNAs are critical for a broad range of biological processes, including T cell homeostasis and activation [16, 18, 30]. Over recent years, accumulating data have also shown that miRNAs are important for modulating CD4^+^ T cell differentiation and plasticity [31]. Recent studies have shown that miRNAs have an active role in the host-pathogen interaction and host immune responses to microorganisms [32]. During parasitic infections, miRNAs are associated with the pathogenesis, drug-resistance, host clearance escape, and host immune response regulation [33]. With respect to miRNAs expression in *echinococcus* species, it has been recently described in relation to its role in host-parasite interaction and future potential use as a diagnostic target [34]. In the present study, miR-374b-5p levels significantly increased after the second week and the fourth week of vaccination with rEg.P29, which indicates that miR-374b-5p may have a transient role in rEg.P29 immunity response. Consequently, the overexpression and inhibition of miR-374b-5p were studied in Th1, Th2, Th17, and Treg cells. We found that overexpression of miR-374b-5p could cause increased IFN-*γ*, IL-2, and IL-17A levels, while inhibition of miR-374b-5p could decrease IFN-*γ* and IL-17A levels. MiR-374b-5p participates in promoting Th1 and Th17 differentiation. Using rEg.P29 as candidate vaccines in this in preclinical study, we identified a key characteristic through which miR-374b-5p exerts a critical immune regulatory mechanism necessary for rEg.P29 protective immunity.

According to the bioinformatics analysis of the database in TargetScan and miRDB, we identified IL-10 as a potential target gene of miR-374b-5p. Overexpression and inhibition of miR-374b-5p could significantly decrease and increase IL-10 mRNA levels. IL-10 is associated with *echinococcosis* infection [27]. It has been suggested that *E. multilocularis* actively governs the immunological orientation of the host through the upregulation of immunosuppressive cytokines, mainly IL-10 and TGF-*β* [29]. Higher levels of IFN-*γ* and reduced expression of IL-10 mRNA levels might be associated with reduced growth of the parasites in vaccinated and treated mice. At the same time, a Th1-oriented response could contribute to the restricted parasite growth of *E. multilocularis* within its intermediate host [19, 20, 24]. This is consistent with recently published data that confirmed the essential role of cellular immunity in controlling *echinococcus multilocularis* infection in humans as well as in mice [24].

IL-10 is an essential anti-inflammatory cytokine mainly produced by Th2 and Tregs [35, 36]. It counteracts CD28 signaling and suppresses the expression of IFN-*γ* and IL-2 [13]. The role of IL-10 in negative regulation of Th1 responses has been highlighted by lethal outcomes of IL-10 knockout or neutralization in toxoplasma or trypanosome mouse infection models [37]. Many immunoregulatory molecules and pathways, most notably those associated with interleukin-10 (IL-10) production, are activated following infection, which can suppress antiparasitic CD4^+^ T cell functions [38]. Recent findings have suggested that IL-10 impairs the Th1 protective response and allows the parasites to survive in *hydatid* patients [24]. Experimental studies in mice supported the possible local immunosuppression mediated by IL-10 as a possible mechanism that helps the parasites in escaping the host cell-mediated response [39, 40]. Our study suggested that higher Th1 and Th17 immune responses were associated with a lower IL-10 mRNA production and miR-374b-5p overexpression, while lower Th1 and Th17 immune responses were associated with a higher IL-10 mRNA production and miR-374b-5p inhibition. IL-10 characterizes immune tolerance in a number of parasitic disease models [41]. The vaccination of rEg.P29 may enhance miR-374b-5p expression induced by a decreased secretion of IL-10, a cytokine typically associated with immunoregulation responses, which appears to limit and ultimately terminate inflammatory responses [42, 43].

In conclusion, miR-374b-5p potentially participates in Th1 and Th17 protective immunity of rEg.P29 vaccination; yet, further studies are needed to identify the signal pathway involved in miR-374b-5p regulating Th1 and Th17 differentiation.

## Data Availability

The data used to support the findings of this study are available from the corresponding author upon request.
